# Ability to Monitor National Responses to the HIV Epidemic “Beyond Viral Suppression”: Findings From Six European Countries

**DOI:** 10.3389/fpubh.2020.00036

**Published:** 2020-03-20

**Authors:** Kelly Safreed-Harmon, Meaghan Kall, Jane Anderson, Natasha Azzopardi-Muscat, Georg M. N. Behrens, Antonella d'Arminio Monforte, Udi Davidovich, Teymur Noori, Jeffrey V. Lazarus

**Affiliations:** ^1^Barcelona Institute for Global Health (ISGlobal), Hospital Clínic, University of Barcelona, Barcelona, Spain; ^2^Public Health England, National Infection Service, London, United Kingdom; ^3^Homerton University Hospital NHS Foundation Trust, Jonathan Mann Clinic, London, United Kingdom; ^4^Department of Health Services Management, Faculty of Health Sciences, University of Malta, Msida, Malta; ^5^Department for Clinical Immunology and Rheumatology, Hannover Medical School, Hanover, Germany; ^6^Institute of Infectious and Tropical Diseases, Department of Health Sciences, ASST Santi Paolo e VCarlo, University of Milan, Milan, Italy; ^7^Department of Infectious Diseases, Research and Prevention, Public Health Service of Amsterdam, Amsterdam, Netherlands; ^8^Department of Infectious Diseases, Amsterdam Infection and Immunity Institute (AIII), Amsterdam UMC, University of Amsterdam, Amsterdam, Netherlands; ^9^European Centre for Disease Prevention and Control, Solna, Sweden

**Keywords:** comorbidity, Europe, health-related quality of life, HIV, indicator, monitoring

## Abstract

**Objective:** With more people living with HIV (PLHIV) ageing into their 50s and beyond in settings where antiretroviral therapy is widely available, non-AIDS comorbidities and health-related quality of life (HRQoL) are becoming major challenges. Information is needed about whether national HIV monitoring programmes have evolved to reflect the changing focus of HIV care.

**Methods:** We created a 56-item English-language survey to assess whether health systems report on common health-related issues for people with HIV including physical and mental health comorbidities, HRQoL, psychosocial needs, and fertility desires. One expert was identified via purposive sampling in each of six countries (Estonia, Italy, the Netherlands, Slovenia, Sweden, and Turkey) and was asked to participate in the survey.

**Results:** Three respondents reported that the current monitoring systems in their countries do not monitor any of four specified aspects of 10 comorbidities including bone loss, cardiovascular disease, and neurocognitive disorders. Two respondents stated that their countries potentially can report on leading causes of hospital admission among PLHIV, and five on leading cases of death. In three countries, respondents reported that there was the ability to report on the HRQoL of PLHIV. In two countries, respondents provided data on the percentage of PLHIV denied health services because of HIV status in the past 12 months.

**Conclusions:** This study identified areas for potential HIV monitoring improvements in six European countries in relation to comorbidities, HRQoL, discrimination within health systems, and other issues associated with the changing nature of the HIV epidemic. It also indicated that some countries either currently monitor or have the ability to monitor some of these issues. There are opportunities for health information systems in European countries to expand the scope of their HIV monitoring in order to support decision-making about how the long-term health-related needs of PLHIV can best be met.

## Introduction

Effective health policy-making and health system management require up-to-date information about people's health-related needs and about health system performance in response to those needs (1). Furthermore, the public reporting of such information promotes accountability by enabling stakeholders to assess the extent to which governments are meeting their health-related obligations (2). In the HIV field, many national governments participate in regional and global monitoring initiatives that require standardised reporting on selected indicators while also collecting data on additional indicators for national monitoring purposes. Decisions about which indicators to include in monitoring can greatly shape national HIV responses since evidence of the need for specific services and commodities can be a major factor in determining how health system resources are allocated.

The highly effective antiretroviral therapy (ART) regimens introduced in the late 1990s have transformed HIV into a manageable long-term condition in the sense that the life expectancy of people who initiate ART at an early stage of infection is close to that of the general population (3). However, as more people living with HIV (PLHIV) age into their 50s and beyond, additional threats to their health and well-being are emerging (4, 5). For multiple reasons, including HIV-mediated chronic inflammation as well as lifestyle factors, PLHIV have higher levels of multimorbidity than the general population (6–8). The disease burden associated with comorbidities is an increasingly prominent concern in HIV clinical care in settings where ART is widely available. A 2018 review found that PLHIV are twice as likely as HIV-negative people to develop cardiovascular disease, and that the global burden of HIV-associated cardiovascular disease has increased three-fold from 1990 to 2015 (9). PLHIV likewise have a higher prevalence of depression, which is underdiagnosed in this population (10). Common causes of hospitalisation among PLHIV in Europe include cardiovascular, respiratory, renal, liver, and psychiatric conditions (11).

**Figure 1:**
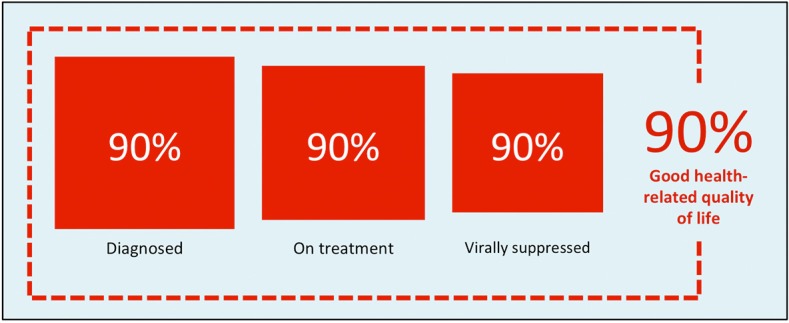
A proposed “fourth 90” target for health-related quality of life [source: Safreed-Harmon et al. (13)].

In 2014, UNAIDS called for international action on an ambitious three-part target: by 2020, 90% of PLHIV were to know their HIV status, 90% of those diagnosed were to be receiving ART, and 90% of those receiving ART were to be virally suppressed (12). The “90-90-90” target has spurred many countries to concentrate resources on increasing diagnosis of HIV and reducing the proportions of diagnosed individuals who do not initiate treatment and do not achieve viral suppression. Meanwhile, there is a dearth of high-level policy guidance addressing what else countries should strive to achieve with regard to the growing number of PLHIV who are likely to live for many years into the future. A “fourth 90” target addressing the health-related quality of life (HRQoL) of PLHIV has been proposed [Figure 1; (5, 13)], but no consensus has emerged regarding how HRQoL should be defined in this context or how progress toward such a target should be measured.

European countries are experiencing the changing HIV care paradigm ahead of many other countries, and European health systems have the opportunity to set an instructive example by adapting their HIV services to reflect a model of care that is suitable for long-term conditions. However, little is known about whether national HIV monitoring programmes in Europe have evolved in accordance with changing needs. Without the right information, health systems will be limited in their efforts to meet new HIV-related health challenges. This study assesses the ability of health systems in six European countries to report on indicators that can speak to the health and psychosocial needs of people who are living with HIV on a long-term basis.

## Methods

### Study Instrument

We identified common health-related issues for people with controlled HIV using an iterative process of desk research and consultation with an expert panel. For the literature review, we used PubMed to identify relevant English-language publications, using the term “HIV” in combination with terms such as “comorbidity,” “Europe,” “epidemiology,” and “health-related quality of life.” We prioritised review articles and large longitudinal cohort studies published after 2010 but did not exclude other sources. We also examined relevant clinical guidelines such as those published by the European AIDS Clinical Society as well as key gray literature sources that were located through internet searches. The expert panel members who advised on the selection of relevant health-related issues to be addressed in the study included European researchers, clinicians, epidemiologists, policymakers, industry representatives, and civil society stakeholders including PLHIV.

We created a 56-item English-language survey to investigate the readiness of national health systems to report on the chosen health-related issues as part of their routine HIV monitoring (Supplementary File). The survey was organised into seven thematic sections: HIV clinical management, comorbidities, health-related quality of life, psychosocial services, discrimination within health systems, preconception planning, and general issues. A number of survey items asked about specific indicators in these areas, instructing respondents to characterise national reporting on these issues by choosing one of four possible responses: (a) national HIV monitoring does include reporting on such an indicator; (b) national HIV monitoring systems collect data that would allow for reporting on such an indicator; (c) national HIV monitoring systems could be easily modified to collect data that would allow for reporting on such an indicator; or (d) national HIV monitoring systems could not be easily modified to collect data that would allow for reporting on such an indicator. In order to keep the survey short enough for respondents to be willing to complete all items, we largely restricted the content to questions such as these about reporting capacity rather than about the actual data being reported. However, some items requested data, e.g., respondents were asked to report leading causes of hospital admission and death among PLHIV and to report the percentage of PLHIV denied health services because of their HIV status.

The survey underwent multiple rounds of revision in response to input from co-authors regarding the topics addressed as well as the structure of the survey questions. Four co-authors reviewed it for clarity and ease of navigation before it was finalised.

### Study Sample

The study group's nine members, who include experts in monitoring, policy, and health system responses to HIV in Europe, were consulted regarding the selection of study countries. The objective was to construct a geographically diverse sample that also included countries with diverse health systems and different levels of robustness in their national HIV monitoring activities. Through this process, Estonia, Italy, the Netherlands, Slovenia, Sweden, and Turkey were selected as study countries. We then identified one expert to serve as the respondent in each country via purposive sampling, drawing on multiple co-authors' networks of contacts in the countries of interest to select this person. The objective was to choose the respondents with the most comprehensive knowledge of their countries' national HIV monitoring mechanisms and resources. There were no other eligibility criteria, and the experts identified included both individuals working directly for government agencies as well as individuals working closely with those agencies. These experts were encouraged to collaborate with other experts in their country to present a comprehensive response.

### Data Collection and Analysis

The survey was administered from 20 April to 30 June 2018 using a Microsoft Word survey document. After data-cleaning, we compiled findings in Microsoft Excel and performed descriptive analyses. For reporting purposes, findings were organised into two domains: (1) ability to report on indicators of interest; and (2) data for selected indicators. In the first domain, a country was considered to be able to report on a specified indicator if the respondent reported that either: (a) national HIV monitoring does include reporting on such an indicator; (b) national HIV monitoring systems collect data that would allow for reporting on such an indicator; or (c) national HIV monitoring systems could be easily modified to collect data that would allow for reporting on such an indicator. We classified a country as not able to report if the respondent reported that national HIV monitoring systems were not currently able and could not be easily modified to collect data that would allow for reporting on such an indicator. Findings relating to comorbidities, health-related quality of life, psychosocial services, discrimination within health systems, and fertility desires are presented in terms of the two domains previously named: reporting ability and data for selected indicators.

## Results

### Participating Study Countries

All six national monitoring experts who were approached about the survey completed it for a 100% response rate. Table 1 describes the HIV epidemiology of the countries included.

**Table 1:** HIV epidemiology in study countries.

	**Estonia**	**Italy**	**The Netherlands**	**Slovenia**	**Sweden**	**Turkey**
European area^a^	East	West	West	Centre	West	Centre
Estimated number of people living with HIV	11,000^b^	127,000^b^	22,900^b^	970^c^	7,700^b^	Unknown
Antiretroviral therapy coverage among people diagnosed with HIV^b^	40%	88%	88%	91%	95%	Unknown
New HIV diagnoses in 2016^a^	229	3,451	745	58	429	2,438

a European Centre for Disease Prevention and Control/WHO Regional Office for Europe (14).

b European Centre for Disease Prevention and Control (15).

c *UNAIDS (16)*.

### Reporting Ability

Regarding ability to report on comorbidities, respondents in three countries (Estonia, Italy, and Turkey) reported that the countries could not monitor any of the four specified aspects (testing/screening offer, uptake, diagnosis, or treatment) of 12 of the 17 comorbidities (Table 2). The comorbidities reported to be most comprehensively monitored by study countries were drug dependence, hepatitis B virus, hepatitis C virus, and tuberculosis. The comorbidities reported to be least comprehensively monitored were anxiety, chronic pain syndrome and depression, all of which could only be monitored by Slovenia and Sweden. There were no comorbidities that all six countries were reported to be able to monitor.

**Table 2:**
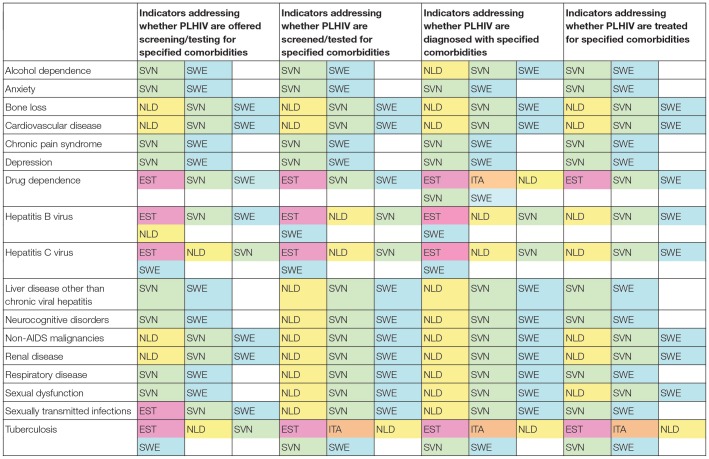
Reported ability of six^*^ European countries to report on indicators for comorbidity screening/testing, diagnosis and treatment in PLHIV.

EST, Estonia; ITA, Italy; NLD, The Netherlands; SVN, Slovenia; SWE, Sweden.

*Turkey was not able to monitor any aspect of any of the comorbidities listed.

According to respondents, two countries had the ability to report on leading causes of hospital admission among PLHIV, while five had the ability to report on leading causes of death (Table 3). Respondents indicated that three countries had the ability to report on the health-related quality of life of PLHIV. Two countries were reported to be able to make modifications to report on the percentage of PLHIV who want to have children, and one, to be able to make modifications to report on the percentage of PLHIV who have an unmet need for preconception planning services.

**Table 3:**
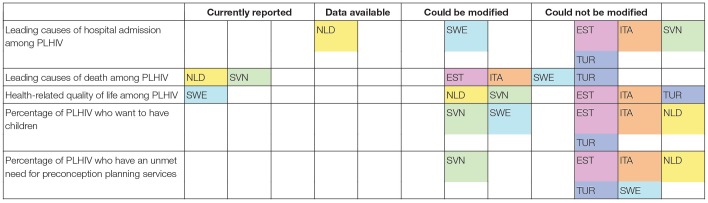
Reported ability of six European countries to report on causes of hospital admission, causes of death, health-related quality of life, and fertility desires in PLHIV (*N* = 6 countries).

Currently reported = National HIV monitoring includes reporting on such an indicator.

Data available = National HIV monitoring systems collect data that would allow for reporting on such an indicator.

Could be modified = National HIV monitoring systems could be easily modified to collect data that would allow for reporting on such an indicator.

Could not be modified = National HIV monitoring systems could not be easily modified to collect data that would allow for reporting on such an indicator.

*EST, Estonia; ITA, Italy; NLD, The Netherlands; SVN, Slovenia; SWE, Sweden; TUR, Turkey*.

Respondents were asked to list up to three indicators used at the national level for monitoring psychosocial service provision (e.g., housing, employment, social support), but none of the respondents reported any psychosocial indicators. Three respondents indicated in comments that no such indicators were used at the national level in their countries. The respondent from Sweden noted that while there was not regular data collection for psychosocial indicators, the Public Health Agency of Sweden had conducted a survey on this issue and planned to repeat the survey approximately every five years.

### Indicator Data

Respondent reporting on the five leading causes of hospital admission and five leading causes of death among PLHIV are shown in Table 4. Respondents in five countries could not report on leading causes of hospital admission, and information for the sixth country, Sweden, reflected the respondent's estimates. In comments from two countries, respondents indicated that data for reporting on this indicator existed: Italy was said to have a national hospital admissions database from which HIV records could be extracted, and the Netherlands was said to have uncoded free text collected in HIV surveillance reports. Respondents in a larger number of countries were able to report data on leading causes of death.

**Table 4:** Leading causes of hospital admission and death among PLHIV in six European countries as reported by respondents^a^.

	**Estonia**	**Italy**	**The Netherlands**	**Slovenia**	**Sweden**	**Turkey**
**Hospital admissions**^**b**^						
1	–	–	–	–	80% late HIV diagnosis	–
2	–	–	–	–	15% ageing-related comorbidities	–
3	–	–	–	–	5% issues related to drug addiction	–
4	–	–	–	–	–	–
5	–	–	–	–	–	–
Year data collected					2017	
**Deaths**^**c**^						
1	–	24% chronic viral hepatitis	25% non-AIDS malignancies	There are only 1 to 5 deaths per year: they are in very late presenters: opportunistic infections, lymphomas and also suicides^d^	Very few deaths. I believe it was 10 during 2017. No specific pattern^d^	–
2	–	11% non-Hodgkin lymphoma	15% cardiovascular disease			–
3	–	12% septicemia	11% AIDS			–
4	–	16% pneumonia	8% non-AIDS infections			–
5	–	10% heart disease	8% lung disease			–
Year data collected		2006–2010	2016^c^			

a Since respondents were asked to report the proportions of hospital admissions and deaths attributable to the top five causes as percentages of total hospital admissions and deaths, percentages may not sum to 100%.

b Reporting for Sweden reflects estimates.

c For The Netherlands, reporting was incomplete at the time the survey was submitted and cause of death was unknown for 12% of deaths.

d Verbatim transcription from survey response.

*“–” indicates that no data were reported*.

Respondents in two countries provided data on the percentage of PLHIV who had been denied health services because of their HIV status in the past 12 months; respondents from the remaining four countries were unable to do so (Table 5).

**Table 5:** Discrimination against PLHIV within health systems in six European countries.

Of all PLHIV, what percentage report being denied health services (including dental care) because of HIV status in past 12 months?^**a**^					
**Estonia**	**Italy**	**The Netherlands**	**Slovenia**	**Sweden**	**Turkey**
–	–	–	10%^b^	–	20%^c^

a Adapted from an indicator in the People Living with HIV Stigma Index (GNP+, ICW, UNAIDS); http://www.stigmaindex.org.

b Source of data unknown.

c Respondent indicated that data were collected in 2011 and were published in Gökengin et al. (17).

*“–” indicates that no data were reported*.

## Discussion

This study in six European countries assessed aspects of health system monitoring that are considered relevant to the care of PLHIV on a long-term basis. Based on input from respondents who are regarded as top HIV monitoring experts in their countries, it found that a number of countries cannot report on indicators for many major comorbidities in PLHIV populations, including highly prevalent comorbidities such as cardiovascular disease and depression. It also identified large monitoring gaps in relation to health-related quality of life, fertility desires, psychosocial services, and discrimination within health systems.

There are multiple processes for monitoring progress against HIV in Europe, with most countries reporting to UNAIDS, the World Health Organization (WHO) and the European Centre for Disease Prevention and Control (ECDC) in addition to conducting further national-level monitoring to inform in-country decision-making. UNAIDS and ECDC reporting influence country-level monitoring activities in Europe, while at the same time, decisions about which indicators to include in multi-country monitoring activities are influenced by what is known about countries' reporting ability. The reported lack of ability of study countries to monitor some issues addressed in our survey suggests a need for country-level stakeholders to re-assess their HIV monitoring priorities as well as a need for regional stakeholders to provide technical support in this area. The reported lack of ability to monitor many comorbidities is particularly disquieting in light of modelling research that indicates that the large comorbidity burden among PLHIV will continue to increase in the coming years (18).

In the interest of capturing as much information as possible, we sought to learn about the monitoring of each comorbidity included in our study in four domains spanning the diagnosis and treatment spectrum: whether PLHIV are offered screening/testing, whether they are screened/tested, whether they are diagnosed and whether they are treated. Three or more countries had the ability to report on all four domains for some comorbidities. While this is a welcome finding, the methodological decision to ask survey respondents to report in terms of the four domains is not meant to imply that health systems should be asked to monitor all four of them. Where health systems do not currently have robust reporting processes in place, a better use of resources would be to focus on collecting data at one stage of the diagnostic process for each comorbidity of interest, e.g., whether PLHIV are screened for cardiovascular disease and whether PLHIV who have been diagnosed with drug dependence receive treatment.

The health-related quality of life of PLHIV has long been a matter of interest for researchers (19), practitioners and community stakeholders, but there is little published information about how this issue has been monitored by health systems. Furthermore, we are not aware of any expert guidance on the use of HRQoL indicators in national HIV monitoring. In 2018, the Dublin Declaration questionnaire (20) included an item about HRQoL for the first time, asking respondent countries to report on whether or not they included HRQoL in their HIV monitoring. Only five of 48 responding countries answered that they did so (personal communication, European Centre for Disease Prevention and Control to Jeffrey V. Lazarus, 5 June 2018). In light of this finding, it is notable that one of the six countries in our study currently carries out reporting on the HRQoL of PLHIV and two more countries said they were able to easily modify their current monitoring systems to carry out such reporting. It may be that there is widespread readiness to incorporate HRQoL into HIV monitoring in the European region, and this is perhaps an opportune time for stakeholders to identify best practices for health information systems to adopt in this regard.

On the other hand, there was little evident capacity to monitor HIV-related discrimination within health systems, which is cause for concern in light of the persistence of such discrimination (21–24) and the impact that it has on the health and well-being of PLHIV (25–27). In 2017, UNAIDS introduced a new indicator for countries to report on in 2018: “Percentage of people living with HIV who report experiences of HIV-related discrimination in health-care settings” (28). This indicator was drawn from an item in the widely used People Living with HIV Stigma Index, a survey developed by a coalition of civil society organisations in collaboration with UNAIDS (29). Our results point to a significant monitoring gap, and in light of the link between HIV-related stigma and poor health outcomes (30), stakeholders are advised to consider whether the Stigma Index indicator selected by UNAIDS should also be added to European regional reporting, which would further encourage national health systems to take up the indicator and collect robust monitoring data.

Given the high level of collaboration and coordination among many European countries in the response to HIV, the use of standardized “fourth 90” HIV care indicators across multiple countries would be beneficial to researchers, policy-makers, and other stakeholders. The optimal way for European countries to choose such indicators would be through a coordinated process that yields recommendations for a small number of new indicators to be used by national governments. In seeking consensus on indicators, stakeholders should consider how to minimise the reporting burden for countries and align data collection with current country-level monitoring activities, particularly in regard to comorbidity monitoring options. With such a large number of comorbidities contributing to the disease burden in PLHIV, the objective should not be to capture information about numerous comorbidities but rather to choose some comorbidities and other indicators as proxies for how health systems are managing comorbidity care in PLHIV overall. Such a process should take into account the possible need to harmonise definitions of some comorbidities such as alcohol dependence and sexual dysfunction.

Efforts to strengthen this aspect of the HIV response might benefit from consideration of lessons emerging from the evolving response to non-communicable diseases (NCDs) since in some regards the HIV and NCD epidemics are challenging health systems in similar ways (31). In a landmark 2018 report on health system responses to NCDs, action points identified by the WHO Regional Office for Europe encompassed issues such as multidisciplinary primary care, people-centred care, and service integration and coordination (32). In the coming years, strategies for monitoring progress on issues such as these are likely to become increasingly relevant to stakeholders in the HIV field.

This study has multiple limitations. The collection of data from only six of the 53 countries in the WHO European Region limits the representativeness of study findings, and a larger number of countries would need to be surveyed to acquire an evidence base for making recommendations about indicators that should be incorporated into national HIV monitoring throughout the region. The recruitment of survey respondents who are regarded as top HIV monitoring experts in their countries may have resulted in selection bias if this strategy led to a lack of representation of countries with HIV monitoring systems that are not sufficiently advanced for any one individual to be regarded as an expert in this area. In study countries where more than one person might be considered suitably knowledgeable about the issues of interest, our choice of experts might be biased by unknown variables. Since only one expert was asked to respond to the survey in each study country, findings directly reflect any gaps in the knowledge of these individuals regarding HIV monitoring in their countries.

The accuracy of the information reported by the survey respondents was not confirmed by other sources, and it may reflect errors or biases on the part of respondents. Biases among respondents also may have influenced some of the estimates and explanations provided in response to survey items that requested data. Country data for leading causes of hospitalisation and death among PLHIV and for discrimination within health systems were not collected using standardised methodologies and definitions. Hence, it is not possible to make meaningful cross-country comparisons. The survey item that asked for the percentage of PLHIV who report being denied health services did not elaborate criteria for what constitutes denial of health services, and thus the outcome measure could have been interpreted in multiple ways.

## Conclusions

Findings from our study indicate that although the existing gaps may be considerable, there are opportunities for health information systems in European countries to expand the scope of their HIV monitoring in order to support decision-making about how the long-term health-related needs of PLHIV can best be met. Scaling up this survey to involve a larger number of European countries would provide stronger evidence regarding how to build on monitoring activities and resources that are already in place. Studies of HIV monitoring capacity are also needed in other regions, including Africa and Asia, where low- and middle-income countries with widespread access to HIV treatment are beginning to face HIV care challenges that similarly call for integrated and people-centred health system responses (31, 33). The larger goals of reorienting monitoring systems to better address the chronic care dimensions of HIV should be to transform HIV care into a more holistic undertaking and to help guide the integration of health system responses to HIV and to other chronic diseases.

## Data Availability Statement

The survey questionnaire and full dataset are available upon request from the authors.

## Author Contributions

JL and KS-H had the idea for this study and developed the study instrument with input from MK and the other co-authors. All authors reviewed and approved the final draft of the article.

### Conflict of Interest

JL received research support and speaking fees from Gilead Sciences, MSD, and ViiV outside of this work. KS-H received support for this work from the HIV Outcomes Beyond Viral Suppression coalition, which is supported by Gilead Sciences and ViiV. GB received honoraria for lecturing and for expert advice from Gilead Sciences, ViiV, Janssen, MSD, and Sandoz outside of this work. JA received honoraria for lecturing and for expert advice from Gilead Sciences, ViiV, and MSD, and also received support for conference attendance from Gilead Sciences outside of this work. The remaining authors declare that the research was conducted in the absence of any commercial or financial relationships that could be construed as a potential conflict of interest.

## Abbreviations

ART: antiretroviral therapy
ECDC: European Centre for Disease Prevention and Control
HRQoL: health-related quality of life
NCDs: non-communicable diseases
PLHIV: people living with HIV
WHO: World Health Organization.
