# Supplementary material for: Ability to Monitor National Responses to the HIV Epidemic “Beyond Viral Suppression”: Findings From Six European Countries
Source: Front Public Health. 2020 Mar 20;8:36. doi: 10.3389/fpubh.2020.00036 (PMC7098908; doi:10.3389/fpubh.2020.00036)
Supplement: Supplementary file 1 [file Data_Sheet_1.PDF]

# Questionnaire to Pilot New HIV Indicators Proposed by the 'HIV Outcomes Beyond Viral Suppression' Research Team

### *Instructions to Pilot Respondents*

Thank you for participating in this pilot survey. We will use the survey results to develop recommendations about how health system monitoring frameworks at the national, regional and global levels can be improved.

We are looking for information about what indicators your national health system currently reports, what data it currently collects as part of monitoring efforts, and what data it could be easily modified to collect. Please carefully read each question as some questions are similar. Please complete as many questions as possible.

Please use the comments field at the end of each question to provide:

- reasons you cannot answer the question (for example, indicator is not relevant to your context or data are available only for specific sub-populations or areas, or from non-nationally representative studies);
- feedback on clarity, definitions and usefulness of the indicators; and
- suggestions for additional indicators for national-level HIV monitoring in relation to the needs of people living with HIV "beyond viral suppression", particularly in regard to comorbidities and health-related quality of life.

## *Country name*

## *Contact information for person completing questionnaire*

First name:
Surname:
Organization:
Job title:
Email:
Phone number (including country code):

# 1 HIV Clinical Management

**1.1 Does national HIV monitoring include reporting on an indicator for the frequency of viral load monitoring (e.g., the number of viral load tests a patient receives in a year)?**

*Please select the **ONE** answer that best describes current national HIV monitoring.*

| | |
|---|---|
| ☐ | National HIV monitoring **does include reporting** on such an indicator. |
| ☐ | National HIV monitoring systems **collect data that would allow for reporting** on such an indicator. |
| ☐ | National HIV monitoring systems **could be easily modified** to collect data that would allow for reporting on such an indicator. |
| ☐ | National HIV monitoring systems **could not be easily modified** to collect data that would allow for reporting on such an indicator. |

Comments to research team:

**1.2 Of all people living with HIV who started antiretroviral therapy in 2012, what percentage are known to have still been on treatment after 60 months?**

%

Comments to research team:

**1.3 Of all people living with HIV and taking antiretroviral therapy, what percentage experienced a treatment interruption as a result of a shortage of one or more required antiretroviral drugs in 2017?**

%

Comments to research team:

**1.4 Of all people living with HIV and taking antiretroviral therapy, what percentage were screened for adherence problems (i.e., for having antiretroviral therapy adherence levels below a defined threshold) in 2017?**

%

Comments to research team:

**1.5 Of all people living with HIV who are known to have been virally suppressed in 2016, what percentage had at least one viral load test documented in 2017?**

%

Comments to research team:

# 2 Comorbidities

**2.1 Does current national HIV monitoring include reporting on indicators that ask whether people living with HIV are offered screening/testing for, are screened/tested for, are diagnosed with or have received treatment for specific comorbidities? For each response cell in the table below, please select the answer that most clearly**

**describes current national HIV monitoring by activating the drop-down menu in the cell and choosing:**

| | |
|---|---|
| Reported | National HIV monitoring **does include reporting** on such an indicator. |
| Collected | National HIV monitoring systems **collect data that would allow for reporting** on such an indicator. |
| Changeable | National HIV monitoring systems **could be easily modified** to collect data that would allow for reporting on such an indicator. |
| Unchangeable | National HIV monitoring systems **could not be easily modified** to collect data that would allow for reporting on such an indicator. |

| **Please select the best match for each of the following.** | **Offered** | **Screened/ tested** | **Diagnosed** | **Treated** |
|---|---|---|---|---|
| 2.1.1 Alcohol dependence | | | | |
| 2.1.2 Anxiety | | | | |
| 2.1.3 Bone loss | | | | |
| 2.1.4 Cardiovascular disease | | | | |
| 2.1.5 Chronic pain syndrome | | | | |
| 2.1.6 Depression | | | | |
| 2.1.7 Drug dependence | | | | |
| 2.1.8 Hepatitis B virus | | | | |
| 2.1.9 Hepatitis C virus | | | | |
| 2.1.10 Liver diseases other than chronic viral hepatitis | | | | |
| 2.1.11 Neurocognitive disorders | | | | |
| 2.1.12 Non-AIDS malignancies | | | | |
| 2.1.13 Renal disease | | | | |
| 2.1.14 Respiratory disease | | | | |
| 2.1.15 Sexual dysfunction | | | | |
| 2.1.16 Sexually transmitted infections (e.g. chlamydia, gonorrhea, syphilis) | | | | |

| | | | | |
|---|---|---|---|---|
| 2.1.17 Tuberculosis | | | | |
| 2.1.18 Other comorbidity: | | | | |

2.1.19 If data about the specific comorbidities identified above are not collected, why are they not, and what are the main barriers to collecting these data?

Comments to research team on all 2.1 questions:

**2.2 Does national HIV monitoring include reporting on an indicator for leading causes of hospital admission among people living with HIV?**

*Please select the **ONE** answer that best describes current national HIV monitoring.*

| | |
|---|---|
| ☐ | National HIV monitoring **does include reporting** on such an indicator. |
| ☐ | National HIV monitoring systems **collect data that would allow for reporting** on such an indicator. |
| ☐ | National HIV monitoring systems **could be easily modified** to collect data that would allow for reporting on such an indicator. |
| ☐ | National HIV monitoring systems **could not be easily modified** to collect data that would allow for reporting on such an indicator. |

Comments to research team:

**2.3 In the most recent calendar year for which hospital admission data were available, what were the five leading causes of hospital admission for people living with HIV? For each cause, report the percentage of hospital admissions attributable to this cause among people living with HIV. (The denominator is the total number of hospital admissions. Thus the percentages attributed to the five leading causes are likely to sum to less than 100%.)**

2.3.1 %

2.3.2 %

2.3.3 %

2.3.4 %

2.3.5 %

2.3.6 Data are from year:

Comments to research team:

**2.4 Does national HIV monitoring include reporting on an indicator for leading causes of death among people with HIV?**

*Please select the **ONE** answer that best describes current national HIV monitoring.*

| | |
|---|---|
| ☐ | National HIV monitoring **does include reporting** on such an indicator. |
| ☐ | National HIV monitoring systems **collect data that would allow for reporting** on |

| | |
|---|---|
| | such an indicator. |
| ☐ | National HIV monitoring systems **could be easily modified** to collect data that would allow for reporting on such an indicator. |
| ☐ | National HIV monitoring systems **could not be easily modified** to collect data that would allow for reporting on such an indicator. |

*If "yes", please continue with question 2.4.1.*
*If "no", please skip to comments.*

2.4.1 Please report the indicator used:

Comments to research team:

**2.5 In the most recent calendar year for which cause of death data are available, what are the five leading causes of death among people with HIV? For each cause, report the percentage of deaths among people diagnosed with HIV attributable to this cause. (The denominator is the total number of deaths. Thus the percentages attributed to the five leading causes are likely to sum to less than 100%.)**

2.5.1 %

2.5.2 %

2.5.3 %

2.5.4 %

2.5.5 %

2.5.6 Data are from year:

Comments to research team:

**2.6 Do national HIV clinical guidelines advise service providers to screen/test people living with HIV at appropriate intervals for the following?**

| Please specify for each of the following | Yes/No answers |
|---|---|
| 2.6.1 Alcohol dependence | Click to Select Yes/No |
| 2.6.2 Anxiety | Click to Select Yes/No |
| 2.6.3 Bone loss | Click to Select Yes/No |
| 2.6.4 Cardiovascular disease | Click to Select Yes/No |
| 2.6.5 Chronic pain syndrome | Click to Select Yes/No |
| 2.6.6 Depression | Click to Select Yes/No |
| 2.6.7 Drug dependence | Click to Select Yes/No |
| 2.6.8 Hepatitis B virus | Click to Select Yes/No |
| 2.6.9 Hepatitis C virus | Click to Select Yes/No |

| | |
|---|---|
| 2.6.10 Liver diseases other than chronic viral hepatitis | Click to Select Yes/No |
| 2.6.11 Neurocognitive disorders | Click to Select Yes/No |
| 2.6.12 Non-AIDS malignancies | Click to Select Yes/No |
| 2.6.13 Polypharmacy | Click to Select Yes/No |
| 2.6.14 Renal disease | Click to Select Yes/No |
| 2.6.15 Respiratory disease | Click to Select Yes/No |
| 2.6.16 Sexual dysfunction | Click to Select Yes/No |
| 2.6.17 Sexually transmitted infections (e.g., chlamydia, gonorrhea, syphilis) | Click to Select Yes/No |
| 2.6.18 Tuberculosis | Click to Select Yes/No |

Comments to research team:

# 3 Health-Related Quality of Life

## 3.1 Does national HIV monitoring include reporting on one or more indicators that address the health-related quality of life of people living with HIV?

*Please select the **ONE** answer that best describes current national HIV monitoring.*

| | |
|---|---|
| ☐ | National HIV monitoring **does include reporting** on such an indicator. |
| ☐ | National HIV monitoring systems **collect data that would allow for reporting** on such an indicator. |
| ☐ | National HIV monitoring systems **could be easily modified** to collect data that would allow for reporting on such an indicator. |
| ☐ | National HIV monitoring systems **could not be easily modified** to collect data that would allow for reporting on such an indicator. |

*If National HIV monitoring does include reporting on such an indicator, please continue with 3.1.1 – 3.1.4. If such an indicator is not reported, please skip to comments.*

3.1.1 What indicator or indicators are used?

3.1.2 Which tool or index is used to measure quality of life?

3.1.3 Does monitoring compare the quality of life of people living with HIV to the quality of life of the general population?

Click to Select Yes/No

3.1.4 When were quality-of-life monitoring data last collected? (Year)

Comments to research team on all 3.1 questions:

# 4 Psychosocial Services

## 4.1 Please describe key indicators and sources of data used at the national level to determine whether services are being provided to meet specific psychosocial needs of people living with HIV in your country (e.g. housing, legal, employment, mental health and social support services).

4.1.1 Indicator 1:

Sources of data used to respond to indicator 1:

4.1.2 Indicator 2:

Sources of data used to respond to indicator 2:

4.1.3 Indicator 3:

Sources of data used to respond to indicator 3:

Comments to research team:

# 5 Stigma and Discrimination within Health Systems

## 5.1 Of all people living with HIV, what percentage report being denied health services (including dental care) because of HIV status in past 12 months?[1]

%

Comments to research team:

# 6 Sexual and reproductive health

## 6.1 Does national HIV monitoring include reporting on an indicator for the percentage of people living with HIV who have an unmet need for contraception?

*Please select the **ONE** answer that best describes current national HIV monitoring.*

| | |
|---|---|
| ☐ | National HIV monitoring **does include reporting** on such an indicator. |
| ☐ | National HIV monitoring systems **collect data that would allow for reporting** on such an indicator. |
| ☐ | National HIV monitoring systems **could be easily modified** to collect data that would allow for reporting on such an indicator. |
| ☐ | National HIV monitoring systems **could not be easily modified** to collect data that would allow for reporting on such an indicator. |

[1] Adapted from an indicator in The People Living with HIV Stigma Index (GNP+, ICW, UNAIDS); http://www.stigmaindex.org).

Comments to research team:

**6.2 Does national HIV monitoring include reporting on an indicator for the percentage of people living with HIV who want to have children?**

*Please select the **ONE** answer that best describes current national HIV monitoring.*

| | |
|---|---|
| ☐ | National HIV monitoring **does include reporting** on such an indicator. |
| ☐ | National HIV monitoring systems **collect data that would allow for reporting** on such an indicator. |
| ☐ | National HIV monitoring systems **could be easily modified** to collect data that would allow for reporting on such an indicator. |
| ☐ | National HIV monitoring systems **could not be easily modified** to collect data that would allow for reporting on such an indicator. |

Comments to research team:

**6.3 Does national HIV monitoring include reporting on an indicator for the percentage of people living with HIV who have an unmet need for preconception planning services?**

*Please select the **ONE** answer that best describes current national HIV monitoring.*

| | |
|---|---|
| ☐ | National HIV monitoring **does** include reporting on such an indicator. |
| ☐ | National HIV monitoring systems **collect data that would allow** for reporting on such an indicator. |
| ☐ | National HIV monitoring systems could be **easily modified** to collect data that would allow for reporting on such an indicator. |
| ☐ | National HIV monitoring systems **could not be easily modified** to collect data that would allow for reporting on such an indicator. |

Comments to research team:

# 7 General issues

**7.1 Do you have any further input about issues raised in this survey?**
